# Automated Biomedical Research Laboratories: Development, Current State, and a Roadmap for Adaptation into Shared Research Resources

**DOI:** 10.7171/001c.167609

**Published:** 2026-09-01

**Authors:** Jay W. Fox, Jeffery R. Martens

**Affiliations:** 1 Office for Research University of Virginia Medical Center; 2 Shared Resources VCU Massey Comprehensive Cancer Center; 3 Department of Pharmacology University of Virginia Medical Center

**Keywords:** laboratory automation, ABRLs, self-driving laboratory, cloud laboratory, biofoundry, core facility, shared research resources (SRRs), reproducibility

## Abstract

Automated biomedical research laboratories (ABRLs) integrate robotics, laboratory instrumentation, orchestration software, and increasingly, machine learning and large language models to plan, execute, and interpret experiments with limited human intervention. Over roughly two decades, these systems have progressed from stand-alone liquid handlers and high-throughput screening workcells to remotely operated “cloud” laboratories, synthetic-biology biofoundries, and closed-loop “self-driving” laboratories capable of autonomous hypothesis generation and testing. This review summarizes the development and current state of ABRLs across biomedical domains, proposes a levels-of-autonomy framework, and evaluates which classes of ABRL are most readily adaptable into institutional shared research resources (core facilities). We maintain that standardized high-throughput sample-preparation and screening workcells, remotely accessible cloud platforms, and design–build–test–learn biofoundry services are the strongest near-term candidates for the core model, whereas bespoke self-driving laboratories are better suited to highly specialized cores or consortium-scale infrastructure. We outline a staged adaptation roadmap including needs assessment and governance, pilot deployment, workflow standardization with rigorous validation and quality management, informatics/remote-access and cost-recovery integration, and federation, and we address the workforce, change-management, and biosecurity-governance factors that determine success. Finally, we propose an evaluation framework that extends an eight-domain core performance model with automation-specific indicators for reliability, reproducibility, cost per result, access equity, and responsible use. Thoughtful adaptation of ABRLs offers core facilities a path to higher throughput, improved rigor and data provenance, and broader access, if standardization, workforce development, and governance keep pace.

## Introduction

Shared research resource core facilities, commonly termed core laboratories or “cores,” generate a large and growing share of the data produced in academic biomedical research. By concentrating advanced instrumentation and technical expertise, cores make efficient use of scarce research funds, broaden access to technologies that individual laboratories cannot sustain, and accelerate discovery.^1^ Their value is repeatedly framed in terms of return on institutional investment,^2^ and the field has developed structured approaches for assessing core performance and outcomes.^3^ Cores also occupy a distinctive position in the movement toward strengthening scientific rigor, reproducibility, and transparency; this is because they standardize protocols, curate provenance, and buffer against cognitive bias in experimental design and analysis.^4^

At the same time, biomedical experimentation is becoming more instrument-intensive, more data-rich, and more sensitive to procedural variation. These pressures have driven the emergence of automated biomedical research laboratories (ABRLs), which are platforms that couple robotics and laboratory instrumentation with software control and, increasingly, artificial intelligence so that experiments can be specified digitally and executed with limited manual intervention. The most advanced of these, often described as “self-driving” or autonomous laboratories, close the loop between experimental design, execution, and analysis by selecting subsequent experiments algorithmically to pursue a stated objective.^5,6^

This review has three aims. First, we summarize the development and current state of ABRLs, organizing a heterogeneous landscape with levels of autonomy framework. Second, we assess which classes of ABRL are most amenable to adoption within the shared resource model and why. Third, we propose how such an adaptation might progress operationally, including the validation, financial, workforce, change management, and governance dimensions that determine success, and how it should be evaluated, extending existing core performance practice with automation-specific measures. Throughout, we emphasize implications for institutional and core facility leaders and the biomolecular resource community rather than focus solely on the engineering details of any single platform, given how rapidly this area is evolving.

## Defining Automated Biomedical Research Laboratories (ABRLs) and Levels of Autonomy

We use “ABRL” as an umbrella term for laboratory systems in which experimental steps are executed by programmable hardware under software control, spanning a spectrum from single automated instruments to fully autonomous, learning-driven platforms. It is important to note five overlapping archetypes. First, unit automation comprises stand-alone automated instruments, such as liquid handlers, automated imagers, plate readers, and sequencers that remove manual steps but are directed experiment-by-experiment. Next, integrated robotic workcells connect several instruments through robotic transport and scheduling to run defined pipelines at scale, a model long used for high-throughput screening. Cloud (remote) laboratories present an automated facility over the internet so that users design and submit experiments as code and receive data without physical presence.^7^ Biofoundries apply automation and high-throughput workflows to the design–build–test–learn (DBTL) cycle of engineering biology.^8^ Finally, self-driving or autonomous laboratories add closed-loop decision-making that uses machine learning to choose each successive experiment toward a user-defined goal.^9,10^

Because “automation” and “autonomy” are frequently conflated, we find it important to rank ABRLs by a levels-of-autonomy scheme that is analogous to those proposed in the broader self-driving-laboratory literature.^10,11^ At Level 0, humans perform all steps. At Level 1, discrete instruments are automated but sequenced manually. At Level 2, integrated workcells execute predefined pipelines with human scheduling and interpretation. At Level 3, remote or cloud operation abstracts the physical laboratory behind a programmatic interface, but experimental strategy remains human-directed. At Level 4, the platform closes the loop, proposing and executing successive experiments under algorithmic control with human oversight of goals and safety. At an aspirational Level 5, the system also formulates hypotheses and interprets results with minimal human involvement. These are capabilities that are demonstrated in narrow domains but not yet generalized.^6^ These levels are not a maturity ranking of individual facilities; a well-run Level 2 workcell may deliver more value to a core than a fragile Level 4 prototype.

## Historical Development of ABRLs

Laboratory automation entered biomedical research at scale through pharmaceutical high-throughput screening, where robotic workcells were built to test large compound libraries against biological assays. The intellectual leap from automated execution to automated reasoning came with the “robot scientist.” The platform named Adam, whose closed-loop design was first described in 2004 and whose autonomous discoveries were reported in 2009, was the first machine able to generate functional genomics hypotheses about yeast metabolism and to then test these hypotheses autonomously using laboratory automation. Adam’s conclusions were subsequently confirmed by manual experiments.^5,6^ Adam’s successor, Eve, which was reported in 2015, was designed to make early-stage drug discovery cheaper and faster; rather than using brute-force screening of every compound, Eve used statistics and machine learning to prioritize hits and, in a widely cited result, identified an anticancer compound with potential activity against malaria.^12^ Historically these systems, Adam and Eve appeared roughly a decade before the recent wave of platforms driven by machine learning and language models; yet, they established the template of closed-loop hypothesis generation, automated execution, and complete digital capture of the experimental record that later self-driving laboratories elaborated.

A parallel thread pursued programmable chemistry: a modular robotic system driven by a chemical programming language demonstrated that multistep organic syntheses could be encoded, shared, and re-executed, thus reframing procedures as portable digital artifacts.^13^ Through the late 2010s, community roadmaps articulated a vision of “next-generation experimentation” in which automation, machine learning, and orchestration software combine into self-driving laboratories,^9^ and consensus perspectives set out the requirements, benchmarks, and open challenges for autonomous experimentation.^14^ Concrete closed-loop platforms followed, which included a self-driving laboratory that advanced the trade-off frontier for a materials property with far fewer experiments than conventional approaches^15^ and an autonomous laboratory that combined computational prediction, robotic synthesis, and automated characterization to produce novel inorganic materials.^16^

In the biosciences, two specific developments broadened access beyond well-resourced laboratories. Synthetic biology consolidated its automation around biofoundries, and a Global Biofoundry Alliance was formed to coordinate infrastructure, share standards, and promote the DBTL model across institutions.^8^ Simultaneously, commercial and academic “cloud labs” made a remote, automated workforce available on demand, allowing experiments to be designed and run around the clock without a physical laboratory.^7^ Most recently, large language models have been coupled with laboratory automation: a GPT-4–driven system, Co-Scientist, autonomously designed, planned, and executed experiments, including reaction optimization, by combining literature and documentation search, code execution, and instrument control.^17^ Underlying many of these systems is orchestration software that schedules instruments, manages data, and coordinates the design–execute–analyze loop.^18^

## Current State and Capabilities

Contemporary ABRLs offer four capabilities directly relevant to biomedical research. The first is throughput and continuity. Integrated workcells and cloud platforms operate continuously, decoupling experimental capacity from the availability of individual researchers.^7^ The second is closed-loop optimization. By selecting each experiment algorithmically, autonomous platforms can reach objectives with markedly fewer experiments than exhaustive or intuition-guided search.^10,15^ The third is data provenance and reproducibility. Because protocols are specified as code and every action is logged, ABRLs can capture experimental metadata far more completely than manual work, which align with the rigor and reproducibility goals central to the core community.^4^ The fourth is accessibility. Cloud and biofoundry models lower the barrier for laboratories that lack capital-intensive instrumentation.^7,8^ This democratizing potential is real but conditional, in the sense that remote and cloud access presumes dependable network connectivity, workable institutional data-security arrangements, and a baseline of informatics and protocol-scripting competence among users. Thus, equitable benefit requires investment in training and user support, not only in hardware.

These capabilities are being realized unevenly across biomedical domains. Genomics has been among the most automation-ready, with robotic library preparation and normalization now routine in many sequencing cores. Compound and phenotypic screening remains the archetypal integrated workcell application and the setting in which autonomous prioritization first proved its worth in drug discovery.^12^ Engineering biology has been reorganized around biofoundry DBTL services that automate strain construction and testing.^8^ Protein science, assay development, formulation, and antibody characterization are increasingly amenable to automated, remotely operated execution on cloud platforms.^7^ Materials-oriented autonomous laboratories, though generally outside the biomedical core per se, have produced the clearest demonstrations of end-to-end closed-loop discovery and are thus methodologically instructive.^15–17^

Substantial limitations temper these advances. Autonomous platforms remain narrow: systems that excel at one objective seldom generalize without re-engineering. They can be brittle when confronted with physical exceptions, such as clogs, precipitates, and contamination, that experienced technicians handle routinely.^11,14^ Capital and maintenance costs are high, and interoperability is constrained by proprietary interfaces and the absence of mature standards for exchanging protocols and data. Validation is nontrivial: demonstrating that an automated workflow produces results equivalent to an accepted manual method requires a deliberate and ongoing effort.^4^ Finally, effective ABRLs depend on a workforce that blends laboratory science, software, and instrumentation engineering, all of which are skills that remain in short supply. These constraints shape which systems can realistically be offered as shared resources today.

## Which ABRLs Could Be Adapted into Biomedical Shared Resources?

The shared resource model rewards certain attributes: broad and recurring demand, workflows that can be standardized and quality-controlled, applicability across many investigators, measurable gains in reproducibility, and a plausible cost recovery structure.^1,3^ Judged against these criteria, the ABRL archetypes differ substantially in their near-term fit for the core model (Table 1).

**Table 1.** ABRL archetypes and their near-term suitability for the shared resource (core) model

**Archetype (level)**	**Representative Examples**	**Core-Fit Attributes**	**Near-⁠Term Availability**
Unit automation (L1)	Liquid handlers; automated imagers; plate readers; sequencers	Standardizable; low integration burden	Embed with existing cores
Integrated workcells (L2)	High-throughput screening and library pepartation pipelines	High recurring demand; strong reproducibility gains	Strong
Cloud/remote labs (L3)	Remotely operated "science-as-a service" platforms	Centralized validation; broad multiuser access (assumes connectivity and informatics support)	Strong
Biofoundries (L3/L4)	Design-build-test-learn engineering biology services	Shared standards; critical mass of users needed	Strong where demand exists
Self-driving labs (L4/L5)	Closed-loop, machine-learning-directed autonomous platforms	Objective-specific; harder to generalize and to schedule equitably	Specialized or consortium-scale

Three classes are strong near-term candidates. Integrated high-throughput sample-preparation and screening workcells (Level 2) fit naturally into the existing genomics, screening, and proteomics core portfolio. Their workflows are standardizable, demand is high and recurring, and they yield direct reproducibility benefits. Cloud or remote-access platforms (Level 3) are attractive because a single institutional subscription or on-premises deployment can serve many investigators, including those at smaller institutions, while centralizing method validation and data governance. This “science-as-a-service” position is essentially the core model expressed digitally.^7^ Biofoundry DBTL services (Level 3/4 within a defined scope) fit institutions with a critical mass of engineering biology users and benefit from the shared standards promoted by the Global Biofoundry Alliance.^8^

Bespoke self-driving laboratories (Level 4/5) are the least straightforward to offer as general-purpose cores. Their closed-loop logic is typically aimed at a specific objective, which limits reuse across unrelated projects and complicates transparent and equitable scheduling.^11,14^ Rather than force the laboratories into a general service model, institutions are better served by deploying them as specialized, objective-scoped cores (for example, an autonomous assay optimization or formulation-screening service) or as a consortium-scale infrastructure shared across institutions, an arrangement that is consistent with prior calls for core consolidation to gain efficiency.^19^ Unit automation (Level 1), by contrast, is usually best embedded within existing cores as a productivity enhancement rather than established as a resource in its own right.

## How Adaptation Might Progress

**Figure 1.**
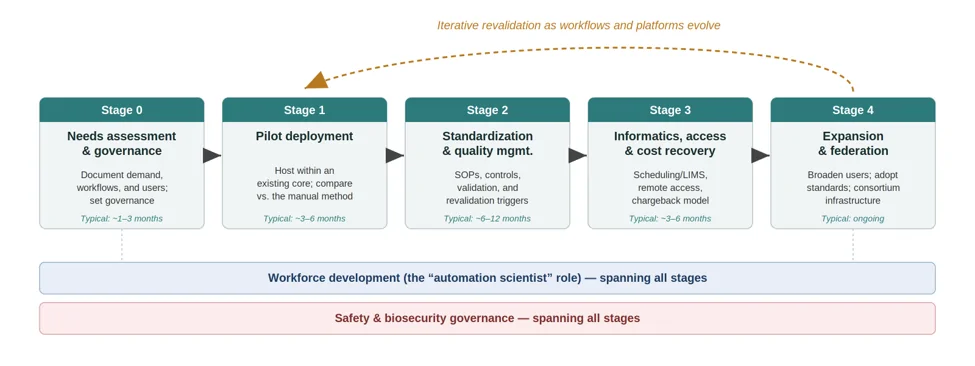
**Staged pathway for adapting an ABRL into a shared research resource.** The schematic depicts five sequential stages: (0) needs assessment and governance; (1) pilot deployment within an existing core; (2) standardization, quality management, and validation; (3) informatics, access, and cost recovery integration; and (4) expansion, federation, and interoperability, with workforce development and safety/biosecurity governance shown as cross-cutting activities spanning all stages. Indicative typical durations are annotated within each stage and will vary with institutional context, platform complexity, and scope. The dashed arrow indicates iterative revalidation as workflows and platforms evolve.

We propose a five-stage adaptation pathway that treats an ABRL not as a purchase but as a service to be designed, validated, and sustained (Figure 1).

### Stage 0: Needs Assessment and Governance.

Before acquisition, leaders should document unmet demand, candidate workflows, and expected users. They should also establish governance for prioritization, data stewardship, and safety. This stage mirrors the strategic planning and stakeholder alignment practices already recommended for building a sustainable core portfolio.^20^

### Stage 1: Pilot Deployment Within an Existing Core

Rather than launching a new facility, an institution can host the platform inside a related core, run a limited pilot for a small set of workflows, and compare automated results against the incumbent manual method.

### Stage 2: Standardization, Quality Management, and Validation

Successful pilots are converted into standard operating procedures embedded in a formal quality management system, with defined acceptance criteria, controls, and revalidation triggers. Validation requires particular rigor because the credibility of an automated shared resource rests on demonstrating that it produces results equivalent to those of an accepted reference method. Concordance studies should be designed rather than improvised: they require samples spanning the analytically relevant range, replication that is sufficient to characterize both within-run and between-run variation, and a sample size powered to detect differences large enough to matter scientifically and not merely to reach statistical significance. The appropriate statistics depend on the data type. For quantitative outputs, programs should use agreement analyses such as Bland–Altman comparison, Deming regression, or the concordance correlation coefficient; for categorical outputs, programs should use sensitivity, specificity, and agreement statistics such as Cohen’s κ.^21^ Acceptance criteria should be domain-specific rather than generic. In genomics, relevant criteria include variant-call concordance, coverage uniformity, and base-quality metrics; in high-throughput screening, assay robustness is commonly judged by the Z′-factor together with signal window and hit-confirmation rates^22^; and in protein or biomarker characterization, criteria typically center on coefficient-of-variation thresholds and recovery against certified reference standards. Responsibility for validation should be shared but clearly assigned: vendors supply platform-qualification data and installation as well as operational qualification, but the core, not the vendor, should own protocol design and the definition of fitness-for-purpose. High-impact or clinically adjacent workflows warrant review by an independent party, such as a quality office or a standing validation committee. Genomics cores have shown that a documented quality management system is both feasible and valuable in the shared resource setting, providing a template for automated services.^23^

### Stage 3: Informatics, Access, and Cost Recovery

The service is integrated with scheduling and laboratory information management systems, programmatic or remote-access interfaces where relevant, and a cost recovery model. Rate-setting for automated services differs from that of conventional cores in both structure and magnitude. The relevant figure is total cost of ownership, which for an automated platform includes not only consumables but also includes robotics and integration, annual service contracts (frequently a substantial fraction of instrument capital per year), software licenses or cloud subscriptions, facility and data-storage costs, and the salary of specialized staff. Single high-end instruments routinely represent six- to seven-figure capital commitments, and integrated or academic cloud-scale facilities have required investments reported in the tens of millions of dollars.^7^ Because automated services typically carry high fixed costs but low marginal costs per sample, their economic models favor volume; recovery models that reflect this, such as subscription or reserved-capacity pricing, tiered internal and external rates, and instrument-time or per-result charges that capture maintenance, software, and staff rather than consumables alone, are more sustainable than the consumable-plus-hourly schemes common to manual cores. Cloud and “science-as-a-service” arrangements (core Contract Research Organization-CRO) convert capital expenditure into operating expenditure, shifting refresh risk to the provider in exchange for recurring fees.^7^ Obsolescence must be planned for explicitly: institutions should budget depreciation and a scheduled refresh, favor modular and upgradable architectures, negotiate upgrade and end-of-support terms with vendors at acquisition, and define sunset criteria for retiring or migrating a service before it becomes a stranded asset. Sound financial management and realistic rate-setting are repeatedly identified as decisive for core sustainability, particularly for capital-intensive services, and prior calls for core consolidation reflect the same high fixed-cost economics.^3,19,24^

### Stage 4: Expansion, Federation, and Interoperability

Mature services can broaden their user base, adopt shared protocol and data standards to enable portability, and, where demand or cost warrants, federate across institutions or contribute to consortium infrastructure.^8,19^ Throughout all stages, two activities must run in parallel rather than simply being deferred: workforce development and safety governance, which will be discussed next.

## Workforce Development: The Automation Scientist

These services depend on people as much as on machines, and the workforce is a fundamental rather than incidental constraint. Automated shared resources require a hybrid professional, an “automation scientist,” who combines wet-laboratory domain knowledge with software and data skills, as well as practical instrumentation and robotics engineering. In practice, the role spans method development and validation, protocol scripting and integration with scheduling and information management systems, routine troubleshooting of hardware and liquid-handling faults, data pipeline and provenance management, and user training. Few individuals arrive with this full complement of skills, so institutions should expect to build this knowledge base deliberately.

Realistic training pathways include cross-training existing core staff by pairing experienced technologists with software and instrumentation mentoring, vendor certification on specific platforms, professional development through organizations such as the Association of Biomolecular Resource Facilities (ABRF) and dedicated graduate education. Carnegie Mellon University’s Master of Science in Automated Science, developed alongside its academic cloud laboratory, is an early example of formal training aimed squarely at this role.^7,25^ Recruitment and retention are complicated by direct competition with industry, which often outbids academic cores for the same hybrid skill set. Useful mitigations include creating genuine career ladders and titles that recognize the role’s technical breadth, competitive and flexibly structured compensation, protected time for professional development, and coappointments that share a specialist across several cores. Because a single expert readily becomes a single point of failure, cross-training and thorough documentation are not luxuries but elements of business continuity.

## Change Management and User Adoption

Automated services change how investigators design and run experiments, and adoption, not installation, determines their success. The principal barriers are behavioral and cognitive rather than technical: investigators must relinquish hands-on control, learn to specify protocols formally, develop trust in results they did not generate by hand in advance, and invest upfront effort in scripting or protocol templating before realizing downstream savings.

Adoption is uneven across user groups. Computationally oriented laboratories, high-throughput and screening users, and trainees, many of whom became comfortable with remote and automated workflows during pandemic-era remote instruction, tend to adopt readily,^7^ whereas laboratories with highly bespoke or artisanal protocols and investigators with long-established manual workflows adopt more slowly. Effective change management therefore pairs technology deployment with deliberate user enabling: template protocols and worked examples that lower the entry cost, onboarding in protocol specification and in interpreting automated quality control and provenance outputs, “power-user” champions embedded in early-adopter laboratories, responsive support such as scheduled office hours, and transparent reporting of validation results to build trust. Framing the core role as collaborative method development rather than mere sample processing helps convert reluctant users into partners.

## How Adaptation Should Be Evaluated

Evaluation should extend, not replace, established core assessment practice. The eight-domain model for core performance includes general management, research and technical staff, financial management, customer base and satisfaction, resource management, communications, institutional impact, and strategic planning, and it provides a validated backbone.^3^ To that backbone, we add automation-specific indicators that are summarized in Table 2, which also proposes a measurement cadence, illustrative targets, and a suggested order of priority so that a launching service can concentrate first on the indicators that most affect credibility and safety. Each indicator should be baselined against the preautomation workflow so that gains and costs are demonstrable rather than assumed.

Five measurement priorities deserve emphasis. First, there is reliability and uptime. Automated services should track instrument availability, failed-run rates, and mean time to recovery because a service that is fast when it works but frequently unavailable does not serve users well. Second, there is reproducibility and rigor. Cores should quantify technical variability, control performance, and concordance with reference methods, reporting these in the same spirit as community rigor and reproducibility efforts.^4,26^ Third, there is cost per result and return on investment. The relevant unit is not the instrument but the validated result that is delivered and assessed against the realistic total cost of ownership.^2^

**Table 2.** Proposed evaluation framework that includes automation-specific indicators layered onto the eight-domain core performance model with suggested measurement cadence, illustrative targets, and priority for a launching service

**Evaluation Dimension**	**Example Indicators**	**Measurement Frequency**	**Illustrative Target/Threshold**	**Priority**
Reliability and uptime	Instrument availablity; failed-run rate; mean time to recovery	Monthly	>95% availability; failed-run rate below a preset celing	High (at launch)
Reproducibility and rigor	Technical CV; control performance; concordance with reference method	Per run and quarterly	CVs within a domain norms: concordance meets predefined acceptance criteria	High (at launch)
Cost per validated result/ROI	Total cost of ownership per result; cost-recovery rate	Quarterly and annual	Full recovery of direct costs; trend toward target subsidy level	Medium
Throughput and turnaround	Samples/experiments per unit time; queue and cycle time	Monthly	Meets or exceeds -re-automation baseline	Medium (baseline at launch)
Access and equity	Utilization by seniority; department; and institution	Semiannual	Broadening user base over time	Medium
User satisfaction	Survey scores; repeat use and rentention	Semiannual	High satisfaction; strong repeat use	Medium
Institutional inpact	Publications; grants enabled; new capabilities created	Annual	Positive and growing contributions	Lower (matures later)
Safety and biosecurity goverance	Protocol-review coverage; access controls; oversight logs	Continuous/per submission	All in-scope submissions reviewed; complete audit trail	High (at launch)

Fourth, there is access and equity. Utilization should be examined across investigator seniority, department, and, for cloud or federated services, across institutions to confirm that automation broadens rather than narrows access.^7^ Fifth, there is safety and biosecurity governance. Because remotely accessible and autonomous platforms lower barriers to executing experiments, evaluation must include oversight of what is run, by whom, and under what review, concerns that are articulated directly in the autonomous laboratory literature.^27^

Practically, we recommend phased key performance indicators aligned to the roadmap. These include pilot-stage metrics focused on method concordance and failure modes; growth-stage metrics focused on utilization, turnaround, and user satisfaction; and mature-stage metrics focused on institutional impact, such as publications, grants enabled, and new capabilities created, together with periodic external review. Standardized, community-level benchmarks would allow cross-facility comparison and would help funders and institutions judge where automated shared resources deliver the greatest value.^14^

## Safety, Biosecurity, and Responsible Use

Because remotely accessible and autonomous platforms lower the practical barriers to executing experiments, they concentrate a governance responsibility that the shared resource community is well positioned to hold. The relevant question is not whether oversight is possible, but how do we adapt existing mechanisms. Institutional biosafety committees, together with established frameworks for dual-use research of concern and for enhanced oversight of certain pathogen research, provide governance structures that can be extended to automated services rather than reinvented.

For cloud and remote platforms, meaningful review is feasible at the point of submission: protocols, where relevant ordered sequences or reagents can be screened before execution, is an approach analogous to the sequence-screening practices adopted by synthetic-DNA providers, supported by user vetting, institutional-affiliation checks, and access tiers calibrated to risk. For autonomous platforms, oversight shifts partly into the system itself, through constraints on permissible objectives, reagents, and reaction or assay steps, and also through human-in-the-loop checkpoints before high-risk actions and through design principles articulated directly in the emerging literature on safe self-driving laboratories.^7,27^ The goal is to balance broad access against responsible use without defaulting to blanket restrictions: tiered, auditable access under institutional oversight preserves the democratizing benefits of these platforms while keeping accountability with identifiable investigators and committees.

## Additional Challenges and Considerations

Several further cross-cutting issues will shape outcomes. Reproducibility and provenance are a genuine opportunity, but they also require disciplined metadata capturing and validation to be realized rather than merely claimed.^4,26^ Standardization and interoperability remain immature; without portable protocols and data formats, institutions risk being locked into single vendors and losing the portability that makes digital protocols valuable.^13^ Financial sustainability is a persistent concern for capital-intensive services and is aggravated by rapid technology turnover, which underscores the need for realistic rate-setting and institutional commitment.^20,24^ Business continuity planning, long recommended for cores, takes on added importance when a single automated platform concentrates the capability that many investigators depend on.^28^

## Conclusion

ABRLs have matured from stand-alone instruments into integrated workcells, cloud platforms, biofoundries, and closed-loop autonomous systems, and they now offer real gains in throughput, reproducibility, and access. Not all of these systems are equally suited to the shared resource models; standardized high-throughput workcells, cloud platforms, and biofoundry services are the most adaptable near-term candidates, while custom-made self-driving laboratories are better suited for deployment as specialized or consortium-scale infrastructure. A staged pathway, anchored in governance, piloting, rigorous validation and quality management, informatics and cost recovery, and eventual federation, can best guide adaptation. An evaluation framework that extends the eight-domain core model with automation-specific measures can make the value of that adaptation demonstrable. Success, however, will depend as much on people and governance as on hardware. When developing the automation scientist workforce, managing the cultural change that automation demands of users and building safety and biosecurity oversight in from the outset is crucial. If the biomolecular resource community pairs these technologies with the standardization, workforce, and governance that cores already do well, automated shared resources can extend the core mission of efficient, rigorous, and broadly accessible science.
